# Supplementary material for: Predicting Causal Relationships from Biological Data: Applying Automated Causal Discovery on Mass Cytometry Data of Human Immune Cells
Source: Sci Rep. 2017 Oct 5;7:12724. doi: 10.1038/s41598-017-08582-x (PMC5629212; doi:10.1038/s41598-017-08582-x)
Supplement: Supplementary file 1 — Supplementary Material [file 41598_2017_8582_MOESM1_ESM.pdf]

Supplementary Information: Predicting Causal Relationships from Biological Data: Applying Automated Causal Discovery on Mass Cytometry Data of Human Immune Cell

Sofia Triantafillou[1,2,+,*], Vincenzo Lagani[1,+], Christina Heinze-Deml[3], Angelika Schmidt[4], Jesper Tegner[4,5], and Ioannis Tsamardinos[1]

[1]Department of Computer Science, University of Crete, Greece
[2]Department of Physical Medicine and Rehabilitation, Northwestern University, Chicago, IL
[3]Seminar for Statistics, ETH Zurich, Switzerland
[4]Unit of Computational Medicine, Center for Molecular Medicine, Department of Medicine Solna, Karolinska Institutet & Karolinska University Hospital, Science for Life Laboratory, Stockholm, Sweden
[5]Biological and Environmental Sciences and Engineering Division, Computer, Electrical and Mathematical Sciences and Engineering Division, King Abdullah University of Science and Technology (KAUST), Saudi Arabia

[*]sofi.triantafyllou@northwestern.edu
[+]these authors contributed equally to this work

Abstract
Learning the causal relationships that define a molecular system allows us to predict how the system will respond to different interventions. Distinguishing causality from mere association typically requires randomized experiments. Methods for automated causal discovery from limited experiments exist, but have so far rarely been tested in systems biology applications. In this work, we apply state-of-the art causal discovery methods on a large collection of public mass cytometry data sets, measuring intra-cellular signaling proteins of the human immune system and their response to several perturbations. We show how different experimental conditions can be used to facilitate causal discovery, and apply two fundamental methods that produce context-specific causal predictions. Causal predictions were reproducible across independent data sets from two different studies, but often disagree with the KEGG pathway databases. Within this context, we discuss the caveats we need to overcome for automated causal discovery to become a part of the routine data analysis in systems biology.

Supplementary Information This document provides supplementary information for the article *Predicting Causal Relationships from Biological Data: Applying Automated Causal Discovery on Mass Cytometry Data of Human Immune Cell*.

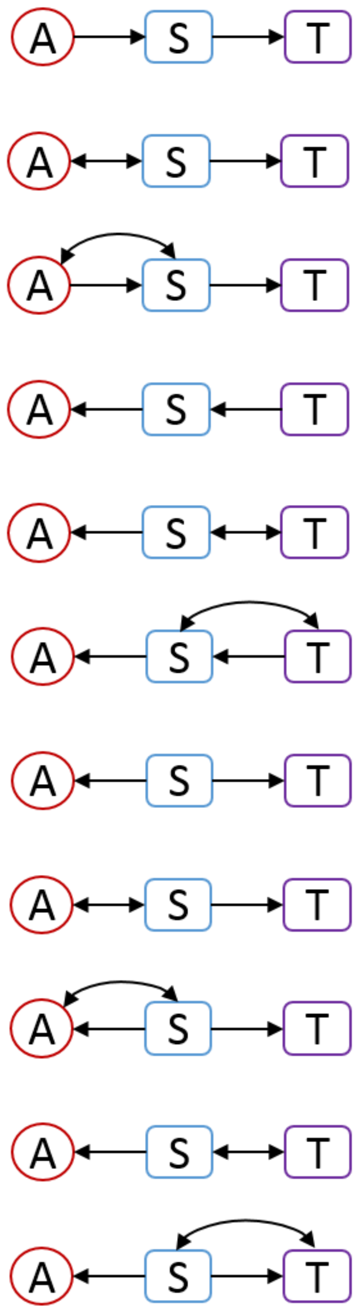

Figure 1. All possible networks for three variables A, S, T that entail A⊥ T|S allowing for confounding and no background knowledge. A bi-directed edge denotes the presence of at least one confounder that causes both endpoints.

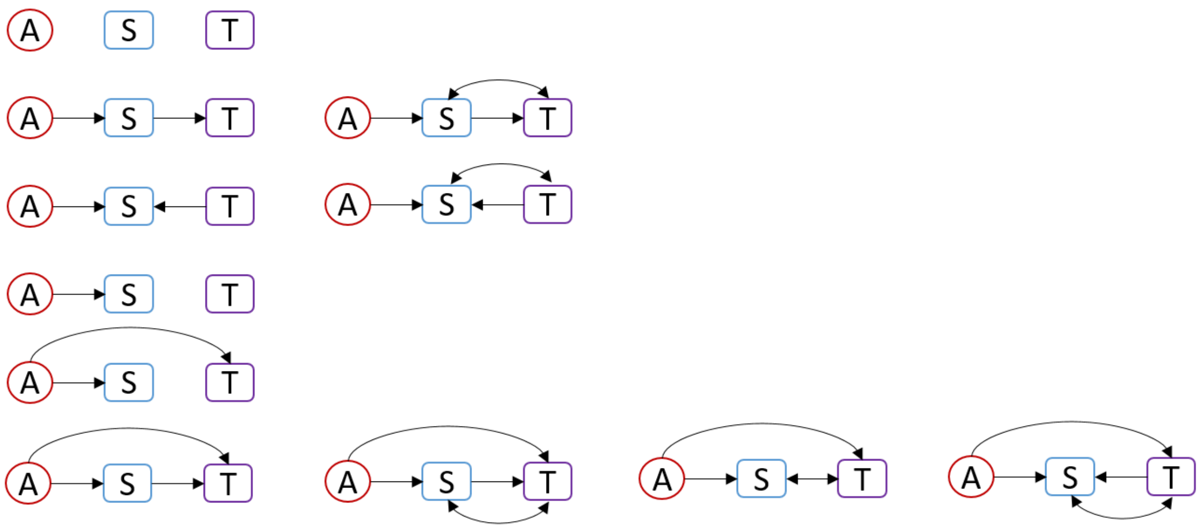

Figure 2. All possible networks for three variables A, S, T where A is known uncaused entity, in the presence of latent confounders. A bi-directed edge denotes the presence of at least one confounder that causes both endpoints.

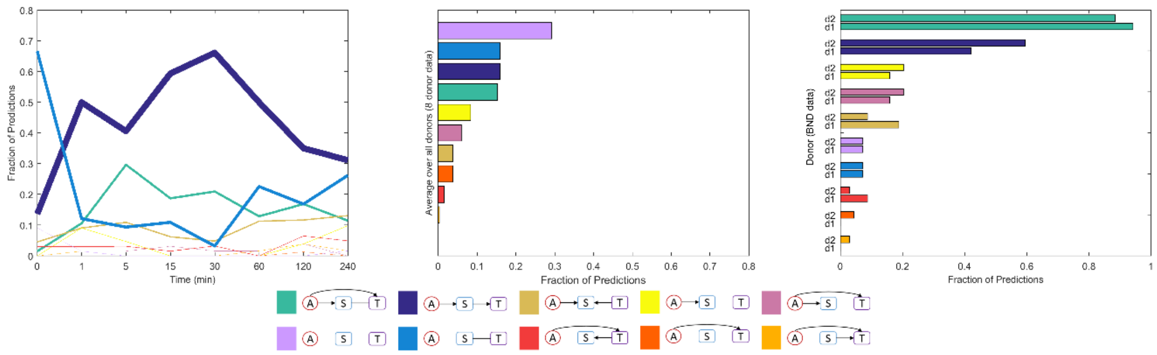

Figure 3. Distribution of highest-scoring networks for CLCD predictions in independent data sets. (left) Different time-points, (middle) different donors, (right) a matching population in bone marrow data from[1] .

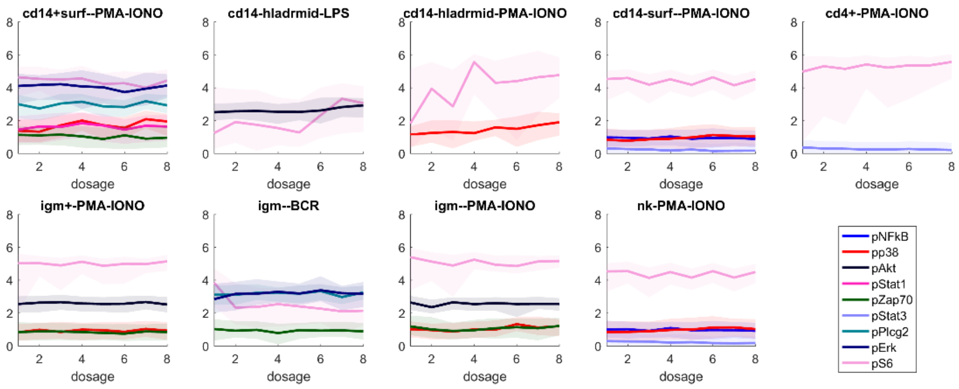

Figure 4. Response of pS6 and its CLCD predicted targets for different Rapamycin dosages

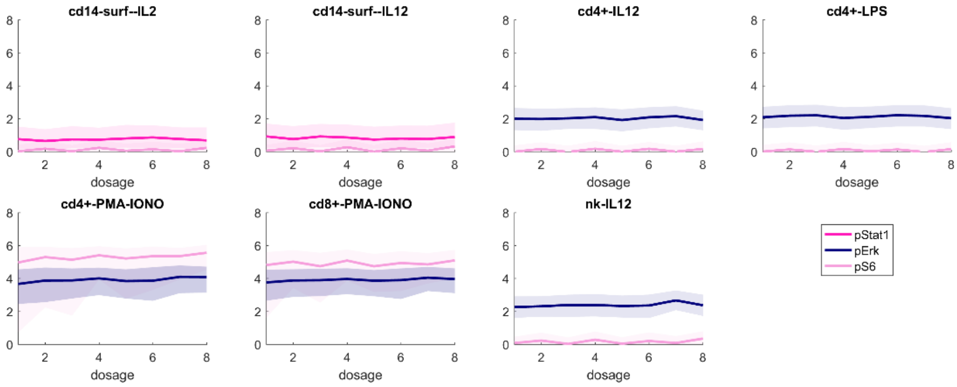

Figure 5. Response of pS6 and its BACKSHIFT predicted targets for different Rapamycin dosages

Supplementary Discussion 1: CLCD hyper-parameters sensibility analysis.

The operation of the Conservative Local Causal Discovery (CLCD) presented in the main paper depends upon three hyper-parameters: the dependence threshold α, the independence threshold β and the number N of replicated datasets where the triplet is confirmed. We perform a sensitivity analysis for these hyper-parameters, assessing whether and how their change would affect our results and conclusion.

We repeated the CLCD analysis by varying α in [0.05, 0.01, 0.005, 0.001], β in [0.1, 0.15, 0.2, 0.25] and setting N to each integer number between 5 and 15.

Supplementary Figures 6 and 7 report the average number of triplets for each value of N and for β set to 0.15 or α set to 0.001, respectively. Both figures shows the same trend: the number of triplets rapidly decreases as the number of replicated datasets increases. The vertical bars show the variations due to changes in α and in β, respectively. Notably, the number of triplets is influenced more heavily by the number N of replicated datasets than by α and β.

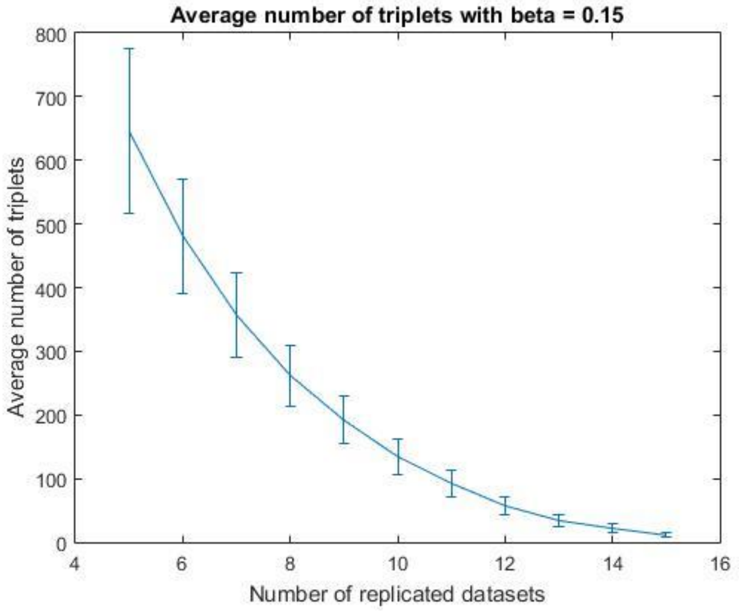

Figure 6. Number of triplets for different number of replicated datasets (x-axis) and beta hyper-parameter set to 0.15. Each point reports the average number of triplets across alpha values, while the vertical bars indicate the respective standard variation.

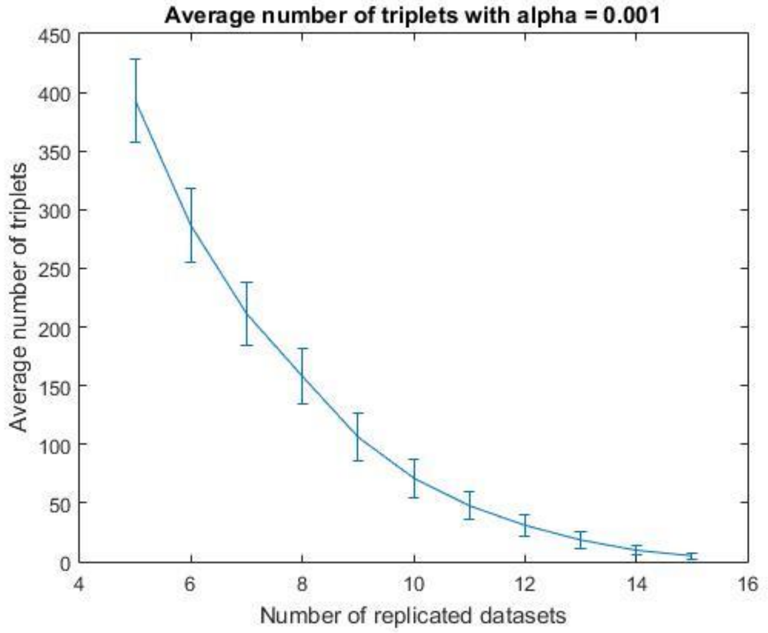

Figure 7: Number of triplets for different number of replicated datasets (x-axis) and alpha hyper-parameter set to 0.001. Each point reports the average number of triplets across beta values, while the vertical bars indicate the respective standard variation.

We then proceed to validate the triplets found for N in [5, 7, 10, 12] against the KEGG databases with the same procedure described in the main text, setting α and β to the same values used in the main paper (0.001 and 0.15, respectively). The results are reported in Supplementary Figure 8 to Figure 11:

it is possible to observe that changing the number of replicated datasets does not remarkably alter the trends in the validation results, neither the final conclusions.

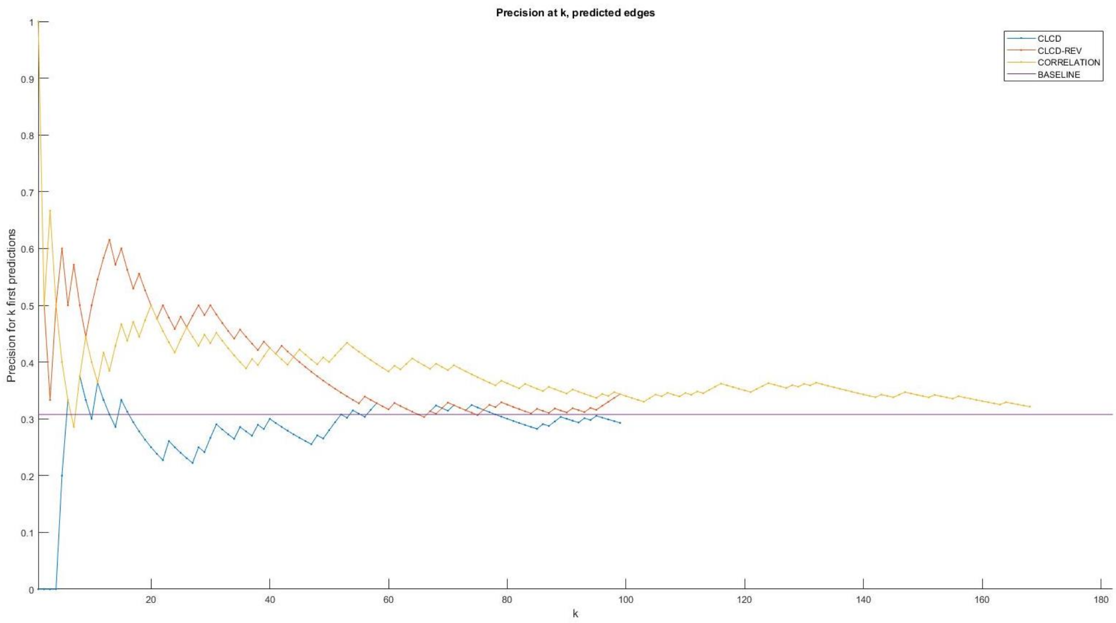

Figure 8: Precision of CLCD predictions in KEGG human pathways for number of replicated datasets set to 5. Details as in Fig.6 of the main text.

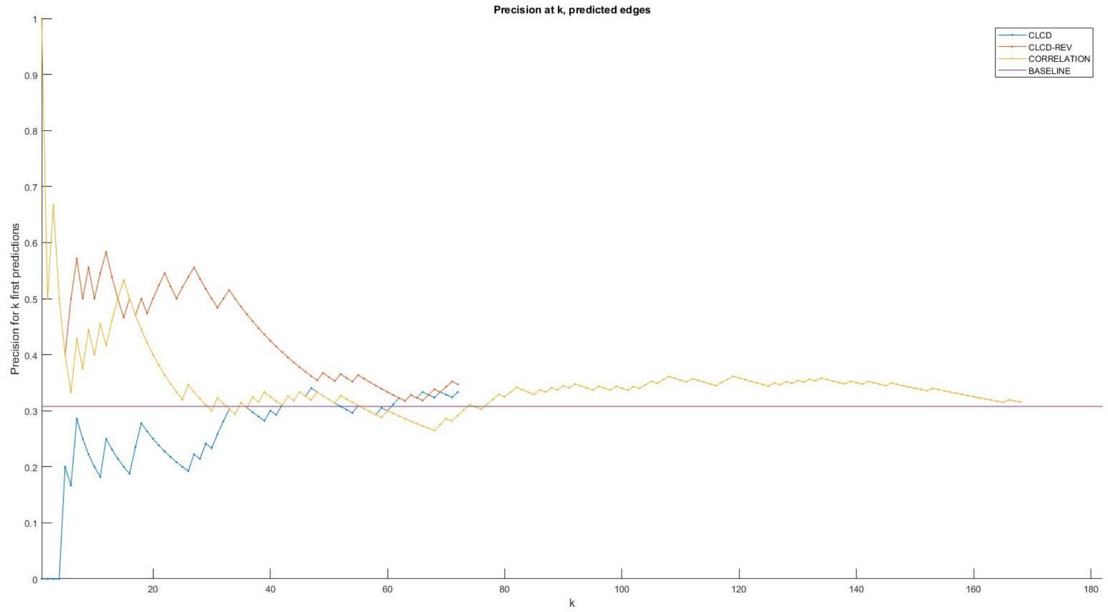

Figure 9: Precision of CLCD predictions in KEGG human pathways for number of replicated datasets set to 7. Details as in Fig.6 of the main text.

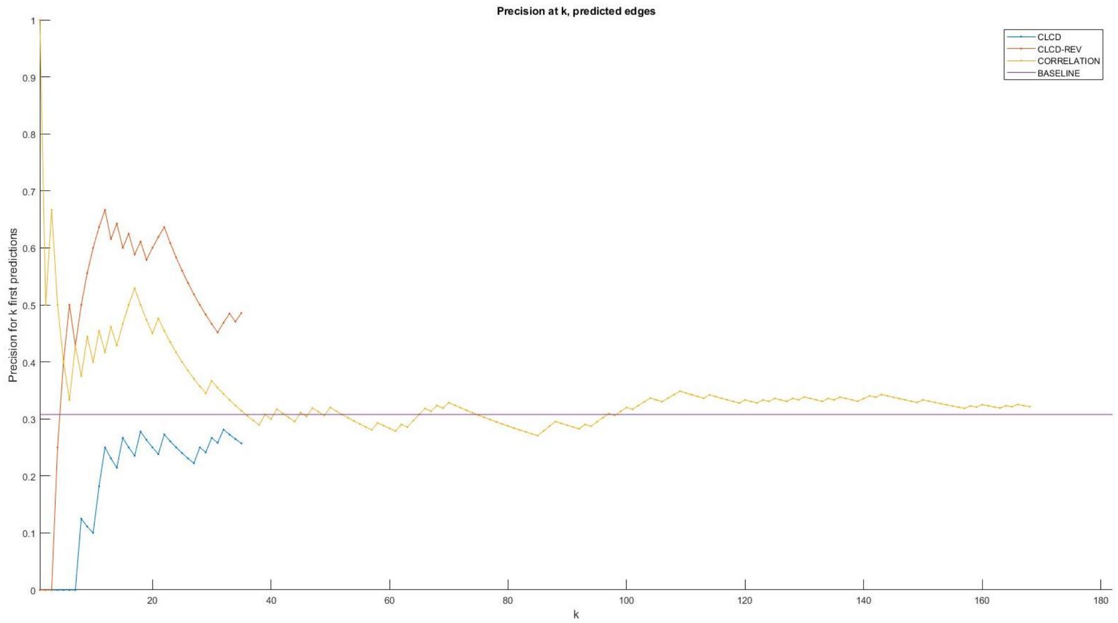

Figure 10: Precision of CLCD predictions in KEGG human pathways for number of replicated datasets set to 10 (as in the main analysis). Details as in Fig.6 of the main text.

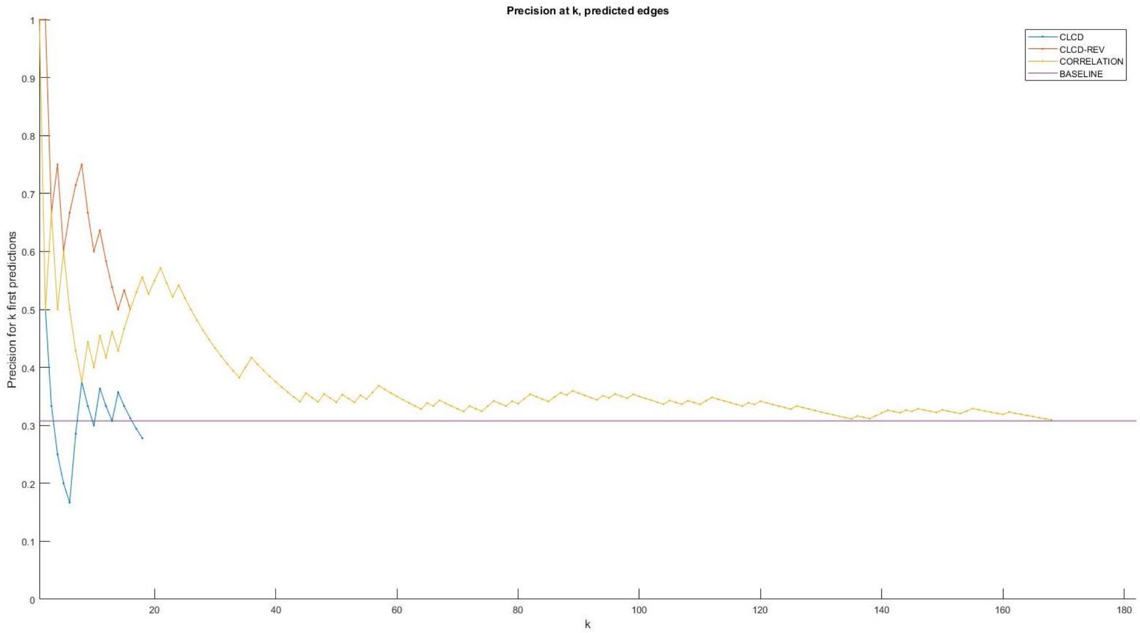

Figure 11: Precision of CLCD predictions in KEGG human pathways for number of replicated datasets set to 12. Details as in Fig.6 of the main text.

Table 1. Proteins and activators measured in[1]and[2].

Functional proteins		Activators	
Bodenmiller et al. (2012)	Bendall et al. (2011)	Bodenmiller et al. (2012)	Bendall et al. (2011)
pNkfb	pNkfb	BCR	BCR
pp38	pp38	GCSF	GCSF
pStat5	pStat5	IFNa	IFNa
pAkt		LPS	LPS
pStat1		PMA-Iono	PMA-Iono
pSHP2	pSHP2	PVO4	PVO4
pZap70/pSyk	pZap70/pSyk	IFN-g	
pSlp76/BLNK	pSlp76/BLNK	IL-2	
pBtk	pBtk	IL-3	IL-3
pPlcg2	pPlcg2	IL-12	
pStat3	pStat3	GMCSF	GMCSF
pErk	pErk		IL-7
pLat			SCF
pS6	pS6		TNFα
	Ki67		TPO
	pCrkL		Flt3L
	pMAPKAPK2		
	IkBa		
	pH3		
	pSrcFK		
	pCREB		

Table 2. Gated subpopulations in[1]and[2].

Population	Subpopulations in Bodenmiller et al. (2012)	Subpopulations in Bendall et al. (2011)
**Monocytes**	CD14+HLA-DR-,<br>CD14+HLA-DR^{high}<br>CD14+HLA-DR^{mid}<br>CD14-HLA-DR-<br>CD14-HLA-DR^{high}<br>CD14-HLA-DR^{mid}	CD11b- Monocyte<br>CD11b^{mid} Monocyte<br>CD11b^{hi} Monocyte
**T-cells**	CD4+	Mature CD4+ T<br>Naive CD4+ T
	CD8+	Naive CD8+ T<br>Mature CD8+ T
**NK**	NK	NK
**B-cells**	Igm+<br>Igm-	Pre-B II<br>Mature CD38^{lo} B<br>Pre-B I<br>Mature CD38^{mid} B<br>Immature B
**Dendritic cells**	Dendritic	Plasmacytoid DC

Table 3. CLCD and BACKSHIFT predictions.

Algorithm	Population	Activator	Source	Target
CLCD	CD14+HLA-DR-	pVO4	pSlp76	pStat3
CLCD	CD14+HLA-DR-	pVO4	pPlcg2	pStat3
CLCD	CD14+HLA-DRmid	pVO4	pSHP2	pZap70
CLCD	CD14+HLA-DRmid	pVO4	pSlp76	pZap70
CLCD	CD14+HLA-DRmid	LPS	pp38	pBtk
CLCD	CD14+surf	pVO4	pStat5	pStat3
CLCD	CD14+surf	pVO4	pStat5	pBtk
CLCD	CD14+surf	pVO4	pSHP2	pStat3
CLCD	CD14+surf	pVO4	pSlp76	pp38
CLCD	CD14+surf	pVO4	pSlp76	pStat3
CLCD	CD14+surf	pVO4	pSlp76	pBtk
CLCD	CD14+surf	pVO4	pPlcg2	pZap70
CLCD	CD14+surf	pVO4	pPlcg2	pStat3
CLCD	CD14+surf	pVO4	pPlcg2	pBtk
CLCD	CD14+surf	PMA-IONO	pp38	pPlcg2
CLCD	CD14+surf	PMA-IONO	pErk	pZap70
CLCD	CD14+surf	PMA-IONO	pS6	pp38
CLCD	CD14+surf	PMA-IONO	pS6	pStat1
CLCD	CD14+surf	PMA-IONO	pS6	pZap70
CLCD	CD14+surf	PMA-IONO	pS6	pPlcg2
CLCD	CD14+surf	PMA-IONO	pS6	pErk
CLCD	CD14-HLA-DR-	pVO4	pPlcg2	pZap70
CLCD	CD14-HLA-DR-	pVO4	pPlcg2	pStat3
CLCD	CD14-HLA-DRmid	pVO4	pSlp76	pSHP2
CLCD	CD14-HLA-DRmid	pVO4	pSlp76	pZap70
CLCD	CD14-HLA-DRmid	pVO4	pPlcg2	pZap70
CLCD	CD14-HLA-DRmid	LPS	pp38	pAkt
CLCD	CD14-HLA-DRmid	LPS	pErk	pAkt
CLCD	CD14-HLA-DRmid	LPS	pS6	pAkt
CLCD	CD14-HLA-DRmid	PMA-IONO	pS6	pp38
CLCD	CD14-surf-	pVO4	pSHP2	pStat3
CLCD	CD14-surf-	pVO4	pSlp76	pStat3
CLCD	CD14-surf-	pVO4	pPlcg2	pStat3
CLCD	CD14-surf-	LPS	pErk	pPlcg2
CLCD	CD14-surf-	PMA-IONO	pp38	pStat3
CLCD	CD14-surf-	PMA-IONO	pErk	pZap70
CLCD	CD14-surf-	PMA-IONO	pErk	pStat3
CLCD	CD14-surf-	PMA-IONO	pS6	pNFkB
CLCD	CD14-surf-	PMA-IONO	pS6	pp38
CLCD	CD14-surf-	PMA-IONO	pS6	pStat3
CLCD	CD4+	IL12	pSlp76	pp38
CLCD	CD4+	IL12	pSlp76	pZap70
CLCD	CD4+	GMCSF	pSlp76	pStat3
CLCD	CD4+	LPS	pSlp76	pStat3
CLCD	CD4+	PMA-IONO	pS6	pStat3
CLCD	CD8+	pVO4	pStat5	pStat3
CLCD	CD8+	pVO4	pSHP2	pStat3

CLCD	CD8+	pVO4	pSlp76	pZap70
CLCD	CD8+	pVO4	pPlcg2	pZap70
CLCD	CD8+	pVO4	pPlcg2	pStat3
CLCD	CD8+	PMA-IONO	pp38	pStat3
CLCD	CD8+	PMA-IONO	pErk	pStat3
CLCD	Igm+	pVO4	pZap70	pp38
CLCD	Igm+	pVO4	pPlcg2	pSHP2
CLCD	Igm+	pVO4	pPlcg2	pSlp76
CLCD	Igm+	BCR	pZap70	pAkt
CLCD	Igm+	BCR	pSlp76	pAkt
CLCD	Igm+	BCR	pErk	pAkt
CLCD	Igm+	PMA-IONO	pAkt	pStat1
CLCD	Igm+	PMA-IONO	pS6	pp38
CLCD	Igm+	PMA-IONO	pS6	pAkt
CLCD	Igm+	PMA-IONO	pS6	pZap70
CLCD	Igm-	pVO4	pZap70	pBtk
CLCD	Igm-	pVO4	pSlp76	pSHP2
CLCD	Igm-	pVO4	pPlcg2	pSHP2
CLCD	Igm-	pVO4	pPlcg2	pSlp76
CLCD	Igm-	BCR	pPlcg2	pSlp76
CLCD	Igm-	BCR	pS6	pZap70
CLCD	Igm-	BCR	pS6	pPlcg2
CLCD	Igm-	BCR	pS6	pErk
CLCD	Igm-	PMA-IONO	pS6	pp38
CLCD	Igm-	PMA-IONO	pS6	pAkt
CLCD	Igm-	PMA-IONO	pS6	pZap70
CLCD	NK	pVO4	pStat5	pStat3
CLCD	NK	pVO4	pSHP2	pStat3
CLCD	NK	pVO4	pSlp76	pStat3
CLCD	NK	PMA-IONO	pErk	pStat1
CLCD	NK	PMA-IONO	pErk	pZap70
CLCD	NK	PMA-IONO	pErk	pStat3
CLCD	NK	PMA-IONO	pS6	pNFkB
CLCD	NK	PMA-IONO	pS6	pp38
CLCD	NK	PMA-IONO	pS6	pStat3
BACKSHIFT	CD14-surf-	IL12	pp38	pNFkB
BACKSHIFT	CD14-surf-	Ref	pp38	pNFkB
BACKSHIFT	CD14-surf-	BCR	pStat3	pNFkB
BACKSHIFT	CD14-surf-	GCSF	pStat3	pNFkB
BACKSHIFT	CD14-surf-	GMCSF	pStat3	pNFkB
BACKSHIFT	CD14-surf-	IL12	pStat3	pNFkB
BACKSHIFT	CD14-surf-	IL2	pStat3	pNFkB
BACKSHIFT	CD14-surf-	LPS	pStat3	pNFkB
BACKSHIFT	CD14-surf-	Ref	pStat3	pNFkB
BACKSHIFT	CD14-surf-	LPS	pNFkB	pp38
BACKSHIFT	CD14-surf-	IFNg	pp38	pStat5
BACKSHIFT	CD14-surf-	IFNg	pZap70	pAkt
BACKSHIFT	CD14-surf-	IL12	pZap70	pAkt
BACKSHIFT	CD14-surf-	IL12	pNFkB	pStat1
BACKSHIFT	CD14-surf-	IL2	pNFkB	pStat1

BACKSHIFT	CD14-surf-	LPS	pp38	pStat1
BACKSHIFT	CD14-surf-	IL2	pStat5	pStat1
BACKSHIFT	CD14-surf-	IFNg	pAkt	pStat1
BACKSHIFT	CD14-surf-	Ref	pAkt	pStat1
BACKSHIFT	CD14-surf-	IL2	pStat3	pStat1
BACKSHIFT	CD14-surf-	IL12	pS6	pStat1
BACKSHIFT	CD14-surf-	IL2	pS6	pStat1
BACKSHIFT	CD14-surf-	IL12	pAkt	pZap70
BACKSHIFT	CD14-surf-	IFNg	pp38	pStat3
BACKSHIFT	CD14-surf-	IFNg	pp38	pSlp76
BACKSHIFT	CD14-surf-	IL2	pBtk	pSlp76
BACKSHIFT	CD14-surf-	IFNg	pPlcg2	pSlp76
BACKSHIFT	CD14-surf-	LPS	pSlp76	pBtk
BACKSHIFT	CD14-surf-	BCR	pAkt	pPlcg2
BACKSHIFT	CD14-surf-	GCSF	pAkt	pPlcg2
BACKSHIFT	CD14-surf-	LPS	pAkt	pPlcg2
BACKSHIFT	CD14-surf-	LPS	pStat1	pPlcg2
BACKSHIFT	CD14-surf-	IL12	pZap70	pPlcg2
BACKSHIFT	CD14-surf-	IL2	pZap70	pPlcg2
BACKSHIFT	CD14-surf-	LPS	pZap70	pPlcg2
BACKSHIFT	CD14-surf-	IL12	pSlp76	pPlcg2
BACKSHIFT	CD14-surf-	LPS	pSlp76	pPlcg2
BACKSHIFT	CD14-surf-	pVO4	pSlp76	pPlcg2
BACKSHIFT	CD14-surf-	pVO4	pSlp76	pPlcg2
BACKSHIFT	CD14-surf-	IL2	pBtk	pPlcg2
BACKSHIFT	CD14-surf-	LPS	pErk	pPlcg2
BACKSHIFT	CD14-surf-	IFNg	pp38	pErk
BACKSHIFT	CD14-surf-	LPS	pSlp76	pErk
BACKSHIFT	CD14-surf-	IFNa	pPlcg2	pErk
BACKSHIFT	CD14-surf-	IFNa	pPlcg2	pErk
BACKSHIFT	CD14-surf-	IFNa	pPlcg2	pErk
BACKSHIFT	CD14-surf-	PMA-IONO	pp38	pS6
BACKSHIFT	CD14-surf-	PMA-IONO	pAkt	pS6
BACKSHIFT	CD14-surf-	PMA-IONO	pErk	pS6
BACKSHIFT	CD4+	IL2	pp38	pNFkB
BACKSHIFT	CD4+	LPS	pp38	pNFkB
BACKSHIFT	CD4+	GCSF	pStat3	pNFkB
BACKSHIFT	CD4+	IFNa	pStat3	pNFkB
BACKSHIFT	CD4+	IFNg	pStat3	pNFkB
BACKSHIFT	CD4+	IL12	pStat3	pNFkB
BACKSHIFT	CD4+	IL2	pStat3	pNFkB
BACKSHIFT	CD4+	IL3	pStat3	pNFkB
BACKSHIFT	CD4+	LPS	pStat3	pNFkB
BACKSHIFT	CD4+	PMA-IONO	pStat3	pNFkB
BACKSHIFT	CD4+	GCSF	pNFkB	pp38
BACKSHIFT	CD4+	IL2	pNFkB	pp38
BACKSHIFT	CD4+	IFNa	pStat3	pStat5
BACKSHIFT	CD4+	IFNa	pStat3	pStat5
BACKSHIFT	CD4+	IL2	pStat5	pStat1
BACKSHIFT	CD4+	GCSF	pAkt	pStat1

BACKSHIFT	CD4+	LPS	pStat3	pSHP2
BACKSHIFT	CD4+	IFNa	pStat5	pStat3
BACKSHIFT	CD4+	LPS	pSHP2	pStat3
BACKSHIFT	CD4+	IFNa	pSHP2	pSlp76
BACKSHIFT	CD4+	LPS	pBtk	pSlp76
BACKSHIFT	CD4+	GCSF	pPlcg2	pSlp76
BACKSHIFT	CD4+	IFNa	pPlcg2	pSlp76
BACKSHIFT	CD4+	pVO4	pPlcg2	pSlp76
BACKSHIFT	CD4+	pVO4	pPlcg2	pSlp76
BACKSHIFT	CD4+	IFNa	pNFkB	pBtk
BACKSHIFT	CD4+	IL12	pErk	pBtk
BACKSHIFT	CD4+	GCSF	pNFkB	pPlcg2
BACKSHIFT	CD4+	IFNa	pp38	pPlcg2
BACKSHIFT	CD4+	BCR	pAkt	pPlcg2
BACKSHIFT	CD4+	IL12	pAkt	pPlcg2
BACKSHIFT	CD4+	LPS	pAkt	pPlcg2
BACKSHIFT	CD4+	Ref	pZap70	pPlcg2
BACKSHIFT	CD4+	GCSF	pSlp76	pPlcg2
BACKSHIFT	CD4+	GCSF	pSlp76	pPlcg2
BACKSHIFT	CD4+	pVO4	pSlp76	pPlcg2
BACKSHIFT	CD4+	pVO4	pSlp76	pPlcg2
BACKSHIFT	CD4+	pVO4	pSlp76	pPlcg2
BACKSHIFT	CD4+	GCSF	pErk	pPlcg2
BACKSHIFT	CD4+	GCSF	pPlcg2	pErk
BACKSHIFT	CD4+	IL12	pS6	pErk
BACKSHIFT	CD4+	LPS	pS6	pErk
BACKSHIFT	CD4+	PMA-IONO	pS6	pErk
BACKSHIFT	CD4+	PMA-IONO	pS6	pErk
BACKSHIFT	CD4+	IL12	pNFkB	pLat
BACKSHIFT	CD4+	GCSF	pBtk	pLat
BACKSHIFT	CD4+	pVO4	pBtk	pLat
BACKSHIFT	CD4+	PMA-IONO	pp38	pS6
BACKSHIFT	CD4+	PMA-IONO	pp38	pS6
BACKSHIFT	CD4+	PMA-IONO	pAkt	pS6
BACKSHIFT	CD4+	PMA-IONO	pAkt	pS6
BACKSHIFT	CD4+	PMA-IONO	pStat1	pS6
BACKSHIFT	CD4+	pVO4	pStat1	pS6
BACKSHIFT	CD4+	LPS	pZap70	pS6
BACKSHIFT	CD4+	PMA-IONO	pPlcg2	pS6
BACKSHIFT	CD4+	PMA-IONO	pPlcg2	pS6
BACKSHIFT	CD4+	PMA-IONO	pPlcg2	pS6
BACKSHIFT	CD4+	PMA-IONO	pErk	pS6
BACKSHIFT	CD4+	PMA-IONO	pErk	pS6
BACKSHIFT	CD4+	PMA-IONO	pErk	pS6
BACKSHIFT	CD4+	PMA-IONO	pErk	pS6
BACKSHIFT	CD4+	PMA-IONO	pErk	pS6
BACKSHIFT	CD4+	PMA-IONO	pErk	pS6
BACKSHIFT	CD4+	PMA-IONO	pErk	pS6
BACKSHIFT	CD4+	PMA-IONO	pErk	pS6
BACKSHIFT	CD4+	PMA-IONO	pErk	pS6

BACKSHIFT	CD4+	PMA-IONO	pErk	pS6
BACKSHIFT	CD4+	PMA-IONO	pErk	pS6
BACKSHIFT	CD4+	PMA-IONO	pErk	pS6
BACKSHIFT	CD4+	BCR	pLat	pS6
BACKSHIFT	CD4+	PMA-IONO	pLat	pS6
BACKSHIFT	CD8+	BCR	pStat3	pNFkB
BACKSHIFT	CD8+	GCSF	pStat3	pNFkB
BACKSHIFT	CD8+	GMCSF	pStat3	pNFkB
BACKSHIFT	CD8+	IFNa	pStat3	pNFkB
BACKSHIFT	CD8+	IL12	pStat3	pNFkB
BACKSHIFT	CD8+	IL2	pStat3	pNFkB
BACKSHIFT	CD8+	IL3	pStat3	pNFkB
BACKSHIFT	CD8+	LPS	pStat3	pNFkB
BACKSHIFT	CD8+	PMA-IONO	pStat3	pNFkB
BACKSHIFT	CD8+	Ref	pStat3	pNFkB
BACKSHIFT	CD8+	IFNa	pStat5	pStat1
BACKSHIFT	CD8+	BCR	pStat5	pStat3
BACKSHIFT	CD8+	IFNa	pp38	pSlp76
BACKSHIFT	CD8+	IL3	pBtk	pSlp76
BACKSHIFT	CD8+	IFNa	pPlcg2	pSlp76
BACKSHIFT	CD8+	IL3	pStat5	pPlcg2
BACKSHIFT	CD8+	LPS	pAkt	pPlcg2
BACKSHIFT	CD8+	GMCSF	pSlp76	pPlcg2
BACKSHIFT	CD8+	pVO4	pSlp76	pPlcg2
BACKSHIFT	CD8+	pVO4	pSlp76	pPlcg2
BACKSHIFT	CD8+	GMCSF	pBtk	pPlcg2
BACKSHIFT	CD8+	IL2	pBtk	pErk
BACKSHIFT	CD8+	pVO4	pBtk	pErk
BACKSHIFT	CD8+	IFNg	pPlcg2	pErk
BACKSHIFT	CD8+	IL3	pPlcg2	pErk
BACKSHIFT	CD8+	PMA-IONO	pS6	pErk
BACKSHIFT	CD8+	PMA-IONO	pS6	pErk
BACKSHIFT	CD8+	GMCSF	pErk	pLat
BACKSHIFT	CD8+	IFNg	pErk	pLat
BACKSHIFT	CD8+	LPS	pErk	pLat
BACKSHIFT	CD8+	LPS	pp38	pS6
BACKSHIFT	CD8+	PMA-IONO	pp38	pS6
BACKSHIFT	CD8+	PMA-IONO	pp38	pS6
BACKSHIFT	CD8+	PMA-IONO	pAkt	pS6
BACKSHIFT	CD8+	PMA-IONO	pZap70	pS6
BACKSHIFT	CD8+	GMCSF	pPlcg2	pS6
BACKSHIFT	CD8+	PMA-IONO	pPlcg2	pS6
BACKSHIFT	CD8+	PMA-IONO	pErk	pS6
BACKSHIFT	CD8+	PMA-IONO	pErk	pS6
BACKSHIFT	CD8+	PMA-IONO	pErk	pS6
BACKSHIFT	CD8+	PMA-IONO	pErk	pS6
BACKSHIFT	CD8+	PMA-IONO	pErk	pS6
BACKSHIFT	CD8+	PMA-IONO	pErk	pS6
BACKSHIFT	CD8+	PMA-IONO	pLat	pS6
BACKSHIFT	NK	BCR	pStat3	pNFkB

BACKSHIFT	NK	GMCSF	pStat3	pNFkB
BACKSHIFT	NK	IL2	pStat3	pNFkB
BACKSHIFT	NK	IFNg	pStat5	pp38
BACKSHIFT	NK	IFNg	pp38	pStat5
BACKSHIFT	NK	IFNg	pp38	pStat3
BACKSHIFT	NK	IL3	pBtk	pSlp76
BACKSHIFT	NK	IFNg	pPlcg2	pSlp76
BACKSHIFT	NK	IFNg	pp38	pBtk
BACKSHIFT	NK	BCR	pAkt	pPlcg2
BACKSHIFT	NK	GMCSF	pAkt	pPlcg2
BACKSHIFT	NK	IFNg	pAkt	pPlcg2
BACKSHIFT	NK	IL3	pAkt	pPlcg2
BACKSHIFT	NK	LPS	pAkt	pPlcg2
BACKSHIFT	NK	Ref	pAkt	pPlcg2
BACKSHIFT	NK	GMCSF	pZap70	pPlcg2
BACKSHIFT	NK	IL12	pZap70	pPlcg2
BACKSHIFT	NK	GMCSF	pSlp76	pPlcg2
BACKSHIFT	NK	GMCSF	pSlp76	pPlcg2
BACKSHIFT	NK	pVO4	pSlp76	pPlcg2
BACKSHIFT	NK	pVO4	pSlp76	pPlcg2
BACKSHIFT	NK	GMCSF	pErk	pPlcg2
BACKSHIFT	NK	IL3	pErk	pPlcg2
BACKSHIFT	NK	Ref	pAkt	pErk
BACKSHIFT	NK	IFNa	pPlcg2	pErk
BACKSHIFT	NK	IL3	pPlcg2	pErk
BACKSHIFT	NK	IL12	pS6	pErk
BACKSHIFT	NK	PMA-IONO	pp38	pS6
BACKSHIFT	NK	PMA-IONO	pPlcg2	pS6

References

1. Bendall, S. C. et al. Single-Cell Mass Cytometry of Differential Immune and Drug Responses Across a Human Hematopoietic Continuum. Science 332, 687–696 (2011).

2. Bodenmiller, B. et al. Multiplexed mass cytometry profiling of cellular states perturbed by small-molecule regulators. Nature biotechnology 30, 858–867 (2012).
